# Enabling knowledge translation: implementation of a web-based tool for independent walking prediction after traumatic spinal cord injury

**DOI:** 10.3389/fneur.2023.1219307

**Published:** 2023-12-05

**Authors:** Ramtin Hakimjavadi, Heather A. Hong, Nader Fallah, Suzanne Humphreys, Stephen Kingwell, Alexandra Stratton, Eve Tsai, Eugene K. Wai, Kristen Walden, Vanessa K. Noonan, Philippe Phan

**Affiliations:** ^1^Faculty of Medicine, University of Ottawa, Ottawa, ON, Canada; ^2^Praxis Spinal Cord Institute, Blusson Spinal Cord Centre, Vancouver, BC, Canada; ^3^Division of Neurology, Department of Medicine, Faculty of Medicine, The University of British Columbia, UBC Hospital, Vancouver, BC, Canada; ^4^Division of Orthopaedic Surgery, Department of Surgery, Faculty of Medicine, University of Ottawa, Ottawa, ON, Canada; ^5^International Collaboration on Repair Discoveries (ICORD), University of British Columbia, Vancouver, BC, Canada

**Keywords:** clinical prediction rule, knowledge translation, prognosis, risk calculator, spinal cord injury, web-based tool

## Abstract

**Introduction:**

Several clinical prediction rules (CPRs) have been published, but few are easily accessible or convenient for clinicians to use in practice. We aimed to develop, implement, and describe the process of building a web-based CPR for predicting independent walking 1-year after a traumatic spinal cord injury (TSCI).

**Methods:**

Using the published and validated CPR, a front-end web application called “Ambulation” was built using HyperText Markup Language (HTML), Cascading Style Sheets (CSS), and JavaScript. A survey was created using QualtricsXM Software to gather insights on the application's usability and user experience. Website activity was monitored using Google Analytics. Ambulation was developed with a core team of seven clinicians and researchers. To refine the app's content, website design, and utility, 20 professionals from different disciplines, including persons with lived experience, were consulted.

**Results:**

After 11 revisions, Ambulation was uploaded onto a unique web domain and launched (www.ambulation.ca) as a pilot with 30 clinicians (surgeons, physiatrists, and physiotherapists). The website consists of five web pages: Home, Calculation, Team, Contact, and Privacy Policy. Responses from the user survey (*n* = 6) were positive and provided insight into the usability of the tool and its clinical utility (e.g., helpful in discharge planning and rehabilitation), and the overall face validity of the CPR. Since its public release on February 7, 2022, to February 28, 2023, Ambulation had 594 total users, 565 (95.1%) new users, 26 (4.4%) returning users, 363 (61.1%) engaged sessions (i.e., the number of sessions that lasted 10 seconds/longer, had one/more conversion events e.g., performing the calculation, or two/more page or screen views), and the majority of the users originating from the United States (39.9%) and Canada (38.2%).

**Discussion:**

Ambulation is a CPR for predicting independent walking 1-year after TSCI and it can assist frontline clinicians with clinical decision-making (e.g., time to surgery or rehabilitation plan), patient education and goal setting soon after injury. This tool is an example of adapting a validated CPR for independent walking into an easily accessible and usable web-based tool for use in clinical practice. This study may help inform how other CPRs can be adopted into clinical practice.

## Introduction

Accurate prognostication, defined as the probability of a person developing a particular state of health or outcome over a specific time (1), can enable clinicians to provide appropriate advice and initiate timely patient-centered management and rehabilitation strategies (2, 3). For spinal cord injury (SCI) this is an active area of research (4–6), that can provide crucial evidence to inform the translation of biomedical and health-related research into better patient outcomes (7).

Prognostic studies can be categorized into four distinct but interrelated themes: fundamental prognosis research, prognostic factor research, prognostic model research, and stratified medicine research (7). In prognostic model research, multiple variables (“predictors”) to estimate a patient's prognosis are considered (1). The result is a prognostic model, also known as a clinical prediction rule (CPR), that combines influential variables to predict the risk of future clinical outcomes in patients, and can be used in various settings for clinical, research and health systems planning applications (1).

Despite the proliferation of CPRs in medical literature, their implementation into clinical practice is limited (8). In general, research suggests that only a small minority of published evidence is translated into clinical practice, and this change occurs slowly over nearly two decades (9). There is a wealth of literature describing potential barriers to account for this, including lack of time, skills, and institutional support to implement clinical practice guidelines (10, 11). Barriers specific to the clinical use of CPRs have recently been discussed in the SCI literature (4). Khan et al. suggested that novel CPRs presented in publications need to be made for accessible to end-users (4). This discrepancy between knowledge creation and knowledge application can be framed as an issue with knowledge translation (KT). KT is a dynamic and iterative process that includes synthesis, dissemination, exchange and ethically-sound application of knowledge, and an approach focusing on closing the gaps between knowledge and practice (12, 13). KT supports moving beyond the dissemination of knowledge (i.e., conducting and publishing prognostic model research) and into the actual use of the knowledge (i.e., the adoption and application of CPRs in the clinical setting). Although the field of KT in SCI is in its early stages, initial evidence supports that KT interventions may change clinician behavior and, ultimately, improve patient outcomes (14). For example, with prognosis research, better translation of published CPRs into clinical practice could guide the clinicians' discussions with patients using reliable evidence-based estimates on the course of their condition. This could help address the variability in information provided by spine surgeons to patients, and the resultant uncertainty in patient expectations regarding outcomes (15).

Examples of non-SCI CPRs that have been successfully adopted into clinical settings include: the Nottingham Prognostic Index for the management of breast cancers (16), Framingham Risk Score for estimating the 10-year Cardiovascular Disease Risk (17), and CHADS (congestive heart failure, hypertension, age ≥75 years, diabetes mellitus, and stroke) score to calculate a patient's risk of having a stroke secondary to atrial fibrillation (18). Reasons for the successful implementation of these CPRs into clinical practice are likely multifactorial, including support from leading professionals in the field and the urgency of the clinical need being addressed; however, these examples also support the notion that CPRs that are easy-to-use are more readily incorporated into clinical practice.

How a CPR is used by its end-users (e.g., clinicians, patients, or researchers) is shaped by its presentation format (19). Various model formats exist for CPRs, including regression formulas, nomograms, score charts, and web-based formats, but there is no consensus on the preference of certain formats over others for optimal communication and use (19). A recent trend toward CPRs being presented as web-based calculators has been noted (20), and several online medical calculators exist, such as MDCalc Medical Calculator (https://www.mdcalc.com). MDCalc offers healthcare professionals with a broad range of clinical tools to support decision-making. The calculators are designed in a practical, easy-to-use format that provide concise, targeted, expert-written content. Thus, in anticipation of the ongoing digitalization in healthcare (21), e.g., with electronic decision support systems and electronic medical records (EMRs), such web-based formats could promote the transportability of CPRs (e.g., by integrating with existing hospital systems) and enable the easy availability of predictions at the point-of-care. Further, our groups' experience in developing and validating the International Standards for Neurological Classification of Spinal Cord Injury (ISNCSCI) Algorithm (https://www.isncscialgorithm.com), supports the use of web-based platforms in SCI clinical practice. The ISNCSCI Algorithm (22), developed by the Praxis Spinal Cord Institute in collaboration with the International Spinal Cord Society (ISCoS), is a free, user-friendly, computerized application designed to convert raw ISNCSCI test scores into accurate classification scores for the SCI now using the revised 2019 ISNCSCI scoring rules. The Algorithm was developed to reduce the high error rates in ISNCSCI exam classification, thus supporting SCI education, research and clinical care (23).

The development and validation of CPRs are only the first steps in KT process. For CPRs to be successfully translated into clinical practice and inform decision-making, they need to be available in a format that can be easily adopted by their end-users. For SCI, given that mobility after injury has been cited by patients as one of their top functional recovery priorities (24), a number of studies have published CPRs for the prediction of independent walking ability after injury (25–29). However, to our knowledge, none are in an easy-to-use format to support adoption into clinical practice. In this study, we aimed to develop a web-based calculator, called Ambulation, using the simplified CPR for prognosticating independent walking after traumatic SCI (TSCI) developed by Hicks et al. (25). The specific objectives were to (1) design and develop the web-based calculator, (2) test and pilot Ambulation with a small group of users, and (3) summarize the feedback from the user-survey and website analytics data. Findings from this study may assist others in the development of novel web-based tools for SCI, incorporating additional research findings in SCI CPRs to make them more accessible and useful for clinicians and patients.

## Materials and methods

In alignment with an integrated KT approach (30), Ambulation was designed to bridge the KT gap, taking a validated CPR for independent walking prediction and transforming it into a user-friendly clinical tool. The following sections describe the steps involved in Ambulation's website design, development, implementation, and monitoring process:

- Planning.
- Design and development.
- Delivery.
- Dissemination.
- Maintenance.

### Planning

Ambulation was co-designed with potential end-users to ensure engagement and adoption with the target audience from the start. In total, a core team of seven clinicians and researchers, and 20 other professionals from different disciplines were consulted. This included, persons with lived experience of SCI, communications and marketing professionals, web developers and IT technicians, as well as a privacy lawyer.

### Design and development

We first considered the operating system, supporting software, and hardware available for the targeted primary end-users (i.e., healthcare professionals such as spine surgeons and allied health professionals treating patients with SCI). Because these end-users would typically have access to a computer or smartphone with internet access (e.g., in the physician's office or at the bedside), Ambulation was implemented to operate on a web browser as a front-end web application.

Initially Ambulation was conceptualized as a one-page website. However, to enhance the user experience, the calculator and other supporting information were designed to be divided across the website as individual pages. The graphical user interface for Ambulation was designed to include five pages, each with a simple layout, consisting of a manageable number of actions, and targeting a specific function. These were the Home, Calculation, Team, Contact, and Privacy Policy page. The Home page and Calculation pages included all the essential information and functionality needed to use the calculator for its intended purpose, i.e., inputting patient data to receive a predicted probability based on the CPR. The Privacy Policy, the Team, and the Contact Us pages contained additional information. Where appropriate, pop-up pages would be included to add additional information that the team deemed was important for the user to read before proceeding further. For example, to ensure that the information generated on Ambulation was appropriately used, a pop-up “User Agreement, Disclaimer, and Consent” was included before the user could access the Calculation page. In this pop-up, users would be informed of the calculator's intended purpose and limitations (i.e., advised that the website is only intended to be used as a tool to assist clinicians in understanding how certain clinical variables relate to walking outcome after TSCI).

We developed Ambulation using HyperText Markup Language (HTML; a markup language), Cascading Style Sheets (CSS; a design language), and JavaScript (a programming language). Together, these three foundational tools in web development enabled the formatting, design, and programming of a lightweight front-end web application. We supplemented this with Bootstrap, a free and open-source CSS framework, to ensure a uniform appearance for prose, tables, and form elements across web browsers (e.g., Chrome, Safari, Internet Explorer, Firefox or Edge). As the calculations to be performed on Ambulation (i.e., the predictions provided by the CPR) are entirely reliant on user input, there was no need to develop a backend with a supporting database which would use more sophisticated web development frameworks. This also ensures that any data entered would not be stored or subject to privacy laws.

When implementing the calculation that would enable the user to estimate the probability of walking independently one-year after TSCI, the mathematical equations from the regression model published in Hicks et al. (25) were extracted. Using this CPR, the end-user entered three patient data points: age (dichotomized at the 65-year-old threshold), highest (left or right) ISNCSCI motor score of the L3 myotome (quadriceps femoris muscle), and highest (left or right) ISNCSCI light touch sensory score of the S1 dermatome completed within 15 days of TSCI (31). The calculation is based on the weighted coefficients to generate a total score (range: −10 to 20). The total score is then used in the regression formula to compute the predicted probability (range: 0 to 1).

The regression formula is as follows:


(1) $$\begin{array}{l} \frac{\text{exp }\left(-1.763+0.125\times score\right)}{1+\text{exp }\left(-1.763+0.125\times score\right)} \end{array}$$

where “score” is the total score. The resultant probability is communicated to the end-user, with precision to two decimal places. In addition, we applied the 0.5 cut-off value used by Hicks et al. to translate the probabilities into the functional outcome of interest. That is, if a person's probability of walking is predicted to be ≥0.5, then they are classified as someone likely to have walking ability; if a person's probability of walking is predicted to be <0.5, then they are classified as likely not to have independent walking ability (26). Both the probability (ranging from 0 to 1) and the final outcome (walk or not walk) rendered by the CPR would be communicated to the end-user (i.e., the clinician), providing flexibility to interpret the results and make optimal use of the data to inform their patients and their clinical decision making. Moreover, information about the definition of “independent walking” would be provided in the calculator's output. Independent walking ability was defined according to the Functional Independence Measure (FIM) (32), a standardized tool for measuring disability, and corresponded to a score of 6 (modified independence) or 7 (complete independence) and a mode of locomotion as walk or both walk and wheelchair (26). These details would allow the results to be interpreted in the context of the original study's definition of independent ambulation.

The calculator was tested using manually derived test cases (*n* = 10) created for debugging purposes, i.e., a process of detecting and removing errors in software code that can cause it to behave unexpectedly or crash. If an unexpected output was observed or a revision was made for improved user experience, the code base was checked, and the identified errors or changes were fixed. The test cases were then applied again until accurate generation of all correct responses was ensured.

To supplement the Calculation page, a section with frequently asked questions was developed. These questions aimed to provide more details regarding Ambulation, including who the intended end-user is, how and when the calculator should be used, and how results should be interpreted. Moreover, other content included a cookie notification pop-up, the Privacy Policy page, the Contact Us page and the Team page.

### Delivery

We planned a pilot launch of Ambulation to gather early utilization data and feedback from targeted end-users. We implemented two systems to monitor website utilization and gather ongoing feedback. Google Analytics (GA, https://analytics.google.com/) was used to monitor real-time website utilization starting from the pilot launch (February 7, 2022). GA data included number of users, average time spent, geographic distribution, user activity trends, and application utilization metrics (clicks, desktop vs. mobile, etc.). In addition, a short user-survey was included using Qualtrics^XM^ (http://www.qualtrics.com/). The survey consisted of four key questions regarding Ambulation's design, utility, and comprehensibility, as well as an open text field for any additional feedback (Supplementary Appendix 1). Upon using the calculator, users are presented with the option to provide their feedback by clicking on the survey link and consenting to the survey.

### Dissemination

For the pilot, several strategies were used to disseminate Ambulation. This included peer-to-peer outreach within existing clinical networks, direct emailing to 30 SCI clinicians, and presentations at SCI conferences i.e., the Canadian Spine Society (CSS) 2022 Annual General Meeting, the “Spinal Columns” CSS Newsletter, American Spinal Injury Association (ASIA) 2022 Annual Scientific Meeting, and GF Strong Research Day-Technology in Rehabilitation 2022.

### Maintenance

GA metrics were monitored monthly to observe trends in website utilization data and identify opportunities for quality improvement on an ongoing basis.

### Data analysis

Descriptive statistics were performed to characterize the survey responses and the GA data. Frequencies and proportions were used to analyze categorical data. Responses to open-ended questions were summarized narratively.

## Results

### Ambulation, a web-based calculator

**Figure 1:**
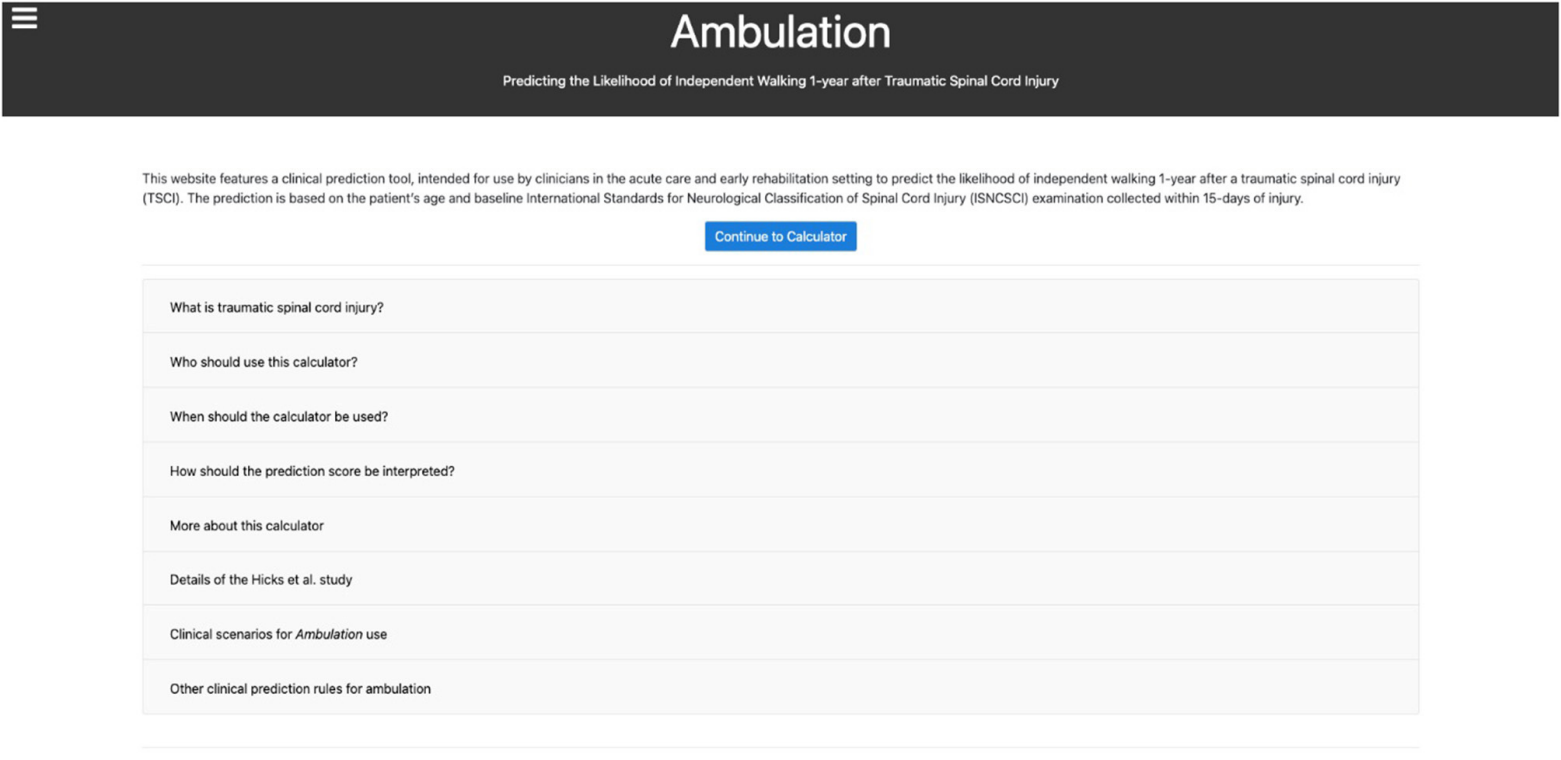
The Ambulation home page. Users are first greeted with a pop-up notification informing them that the website uses cookies and that by continuing they agree to the use of cookies outlined in the Privacy Policy. On this page, a brief description of the web-based calculator is presented, followed by eight “frequently-asked-questions” to important topics before proceeding to the Calculator Page.

**Figure 2:**
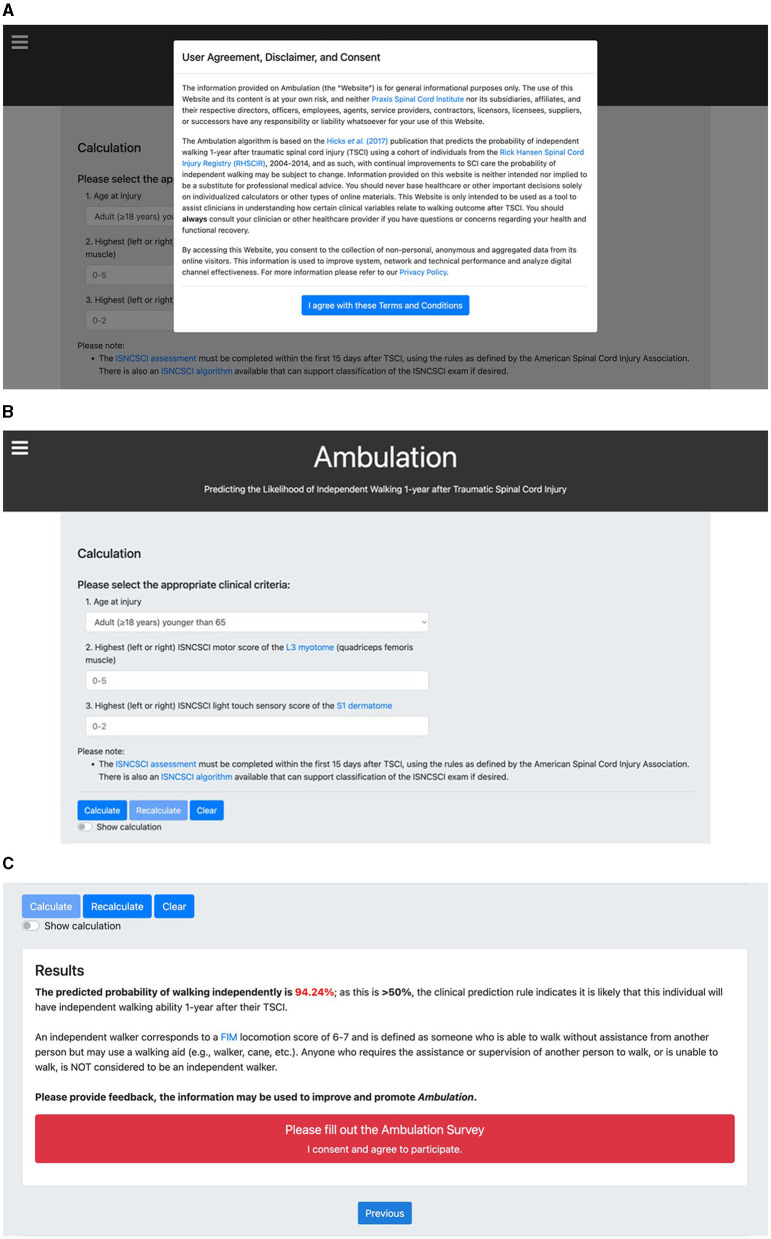
The Ambulation calculator page. **(A)** Upon entering the Calculator Page, a “User Agreement, Disclaimer and Consent” pop-up notification appears. The user must agree to terms and conditions before proceeding to the calculation. **(B)** The calculation is based on the clinical prediction rule (CPR) by Hicks et al. (25). The calculation requires the user to input three patient-related variables: age at injury dichotomized at 65 years old and two items from the ISNCSCI assessment completed within the first 15 days after TSCI. **(C)** The CPR result and interpretation are provided to the user and a link to the feedback survey on Qualtrics is presented.

**Figure 3:**
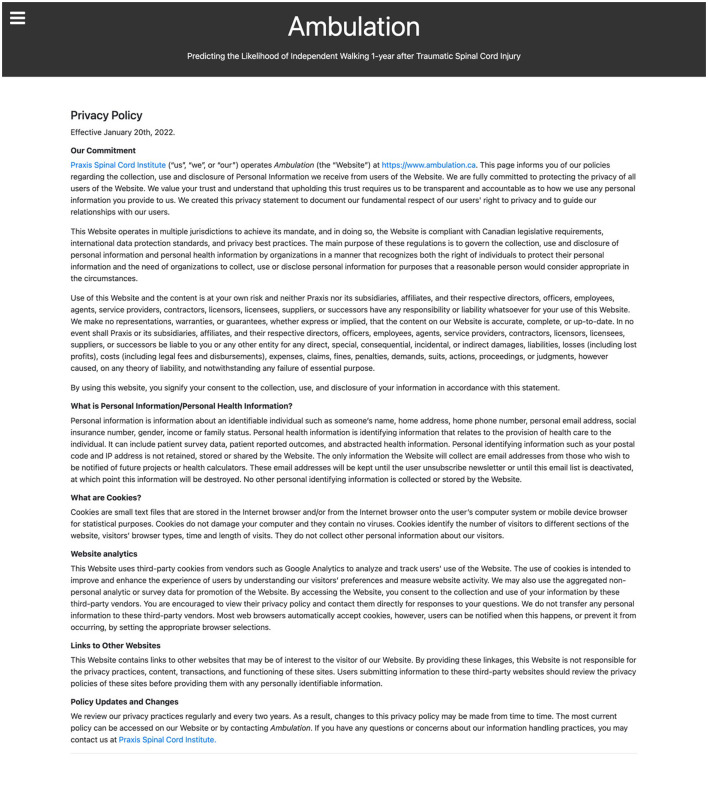
The Ambulation privacy policy page. To comply with local and international data privacy laws, this page describes how the website collects, uses, and manages the personal information received from all users.

**Figure 4:**
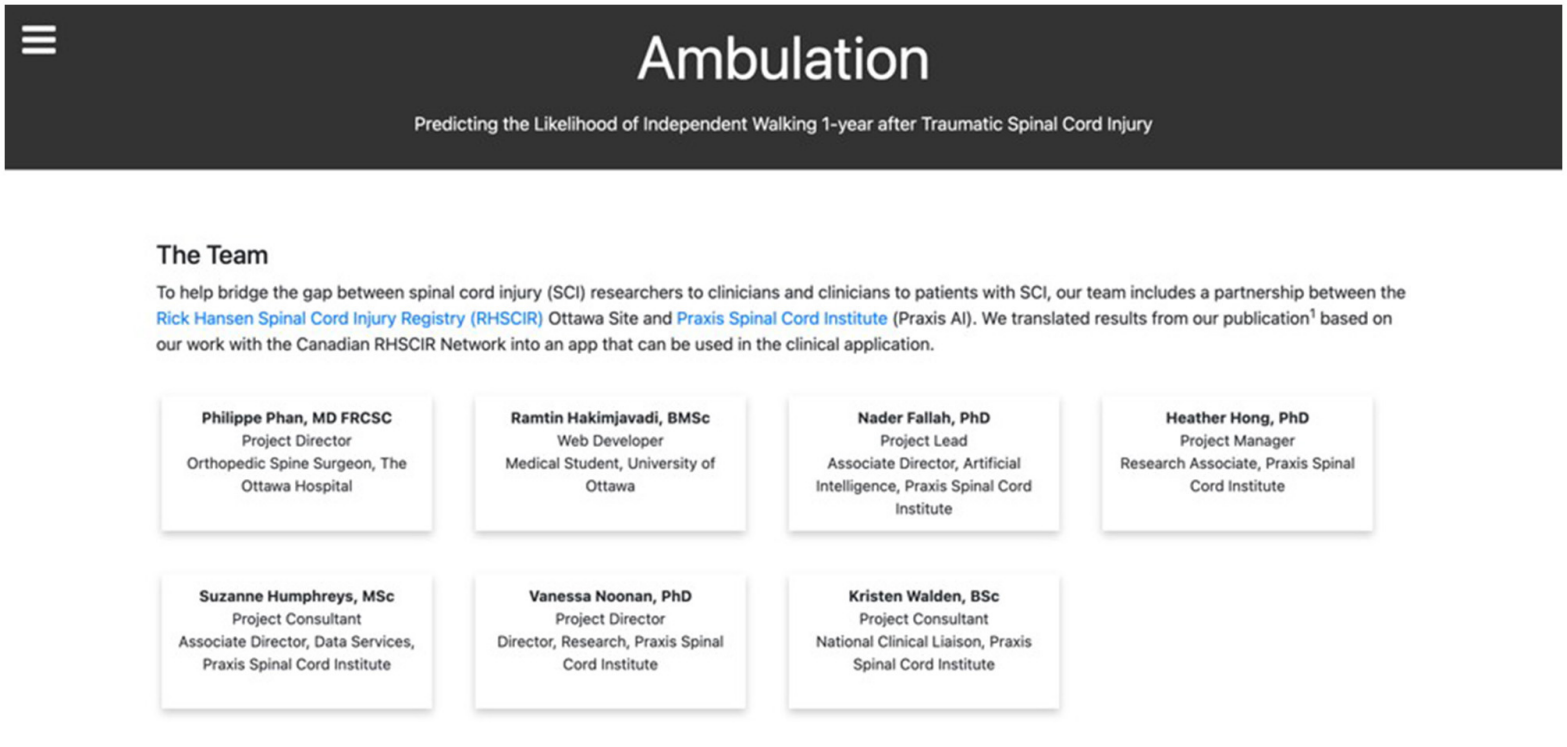
The Ambulation team page. An overview of the development team and their roles.

**Figure 5:**
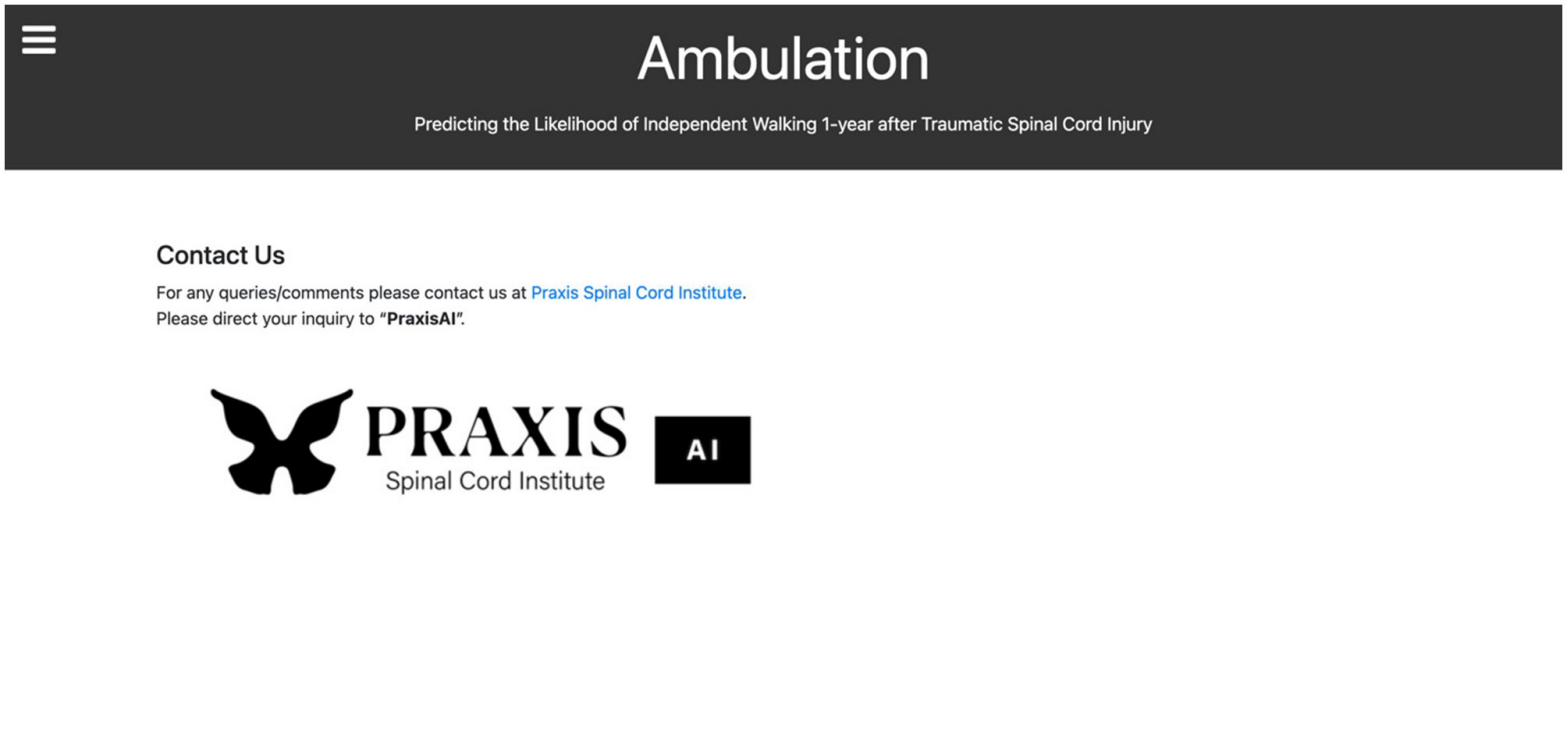
The Ambulation contact us page. For users to easily contact the Ambulation team for any comments or suggestions.

Ambulation was designed and developed as a front-end web site that incorporated the simplified CPR for predicting independent walking ability 1-year after TSCI by Hicks et al. (25). Ambulation underwent 11 revisions to clarify communication and calculator output, improve the design, layout, and site navigation for the end-user. The final version consisted of five pages (Figures 1–**5**). On February 7th, 2022 Ambulation was uploaded onto its own unique domain: http://www.ambulation.ca.

A typical workflow for a user begins with the Home Page (Figure 1). Upon entering, per local and international data privacy laws, the user is informed that the website uses cookies in the form of a pop-up notification. The window appears along the bottom of the webpage without any action from the user. The user is provided with a brief description of the web-based calculator, and the option to proceed to the calculator by clicking “Continue to Calculator.” Additionally, there are eight “frequently-asked-questions” displayed in a drop-down list of responses pertaining to each question. The drop-down feature was intentionally chosen to maintain simplicity and only show information that the user is actively seeking. When proceeding to the calculator, the user encounters a second pop-up with the “User Agreement, Disclaimer, and Consent” (Figure 2A). The user must read and agree to terms before proceeding. Importantly, users are advised that the website is only intended to be used as a tool to assist clinicians in understanding how certain clinical variables relate to walking outcome after TSCI. Further, non-clinician users are advised to always consult their clinician, or other healthcare provider, if they have questions or concerns regarding their health and functional recovery. The calculator requires three input variables (Figure 2B) as described previously. Additionally, on this page links to external resources, such as the ISNCSCI assessment, are provided. These were included to equip the clinician with adequate background information to perform an appropriate assessment of their patient, to ultimately increase the likelihood that accurate patient information is entered into the calculator. If the required input data were entered correctly (i.e. within a range of valid scores), clicking “calculate” will provide the results of the CPR (Figure 2C). Each input parameter has a range of valid scores that are shown to the user. If any input data was entered incorrectly, the user is notified that specific information needs to be changed (e.g., the following field is empty or invalid: motor score L3 must be ≥0 and ≤5) in order for the calculation to be performed. Users are invited to complete a feedback survey each time a calculation is performed. Other options on the calculator include “Recalculate” to update the results if input data were changed, “Clear” to reset input data, or a “Show calculation” option to display how the probability estimates from the CPR are calculated. The Privacy Policy Page (Figure 3), the Team Page (Figure 4), and the Contact Us Page (Figure 5), contain supplementary information that can be sought via links at the bottom of the webpage (Privacy Policy Page) or in the top-left corner (Team Page, Contact Us Page).

In total, 15 external links were included throughout Ambulation, providing additional guidance to the user regarding peer-reviewed publications related to the CPR, information about the ISNCSCI assessment required to use the calculator, or more information about the sponsoring institution (Praxis).

### Survey results

February 7th 2022, Ambulation was piloted by 30 test-users, which included spine surgeons, physiatrists and physiotherapists. Six survey responses were received (20% response rate). Based on question one, using the 5-item Likert scale, most respondents thought Ambulation was easy to navigate, use and understand (Table 1). Most respondents (66.7%) would prefer to use Ambulation on a desktop PC or MacBook, rather than using a smartphone. All six respondents said they would recommend Ambulation to others. When asked to indicate why, responses were: “*Helpful and basic*”; “*Prediction value. Easy to use*”; “*Easy to use and provides great information for discharge planning*”; “*Simple and quick*”; and “*Planning for rehab*.”

**Table 1:** Question 1 survey responses using the 5-item Likert scale ranging from strongly agree to strongly disagree.

	**Strongly agree**	**Somewhat agree**	**Neither agree, nor disagree**	**Somewhat disagree**	**Strongly disagree**	**Total**
Ambulation's website is easy to navigate	5 (83.3%)	0	0	0	1 (16.6%)	6
Ambulation's external website links to other sources, such as the ISNCSCI 2019 publication or ISNCSCI algorithm, are helpful	3 (60.0%)	1 (20%)	0	0	1 (20%)	5
Ambulation's design, layout, color and contrast are visually appealing	3 (60.0%)	1 (20%)	0	0	1 (20%)	5
Ambulation, the calculation page is easy to use	5 (83.3%)	0	0	0	1 (16.6%)	6
Ambulation, the result generated is easy to understand	4 (66.7%)	1 (16.7%)	0	0	1 (16.7%)	6
Ambulation is applicable for clinical use	3 (50%)	1 (16.7%)	1 (16.7%)	0	1 (16.7%)	6
Ambulation's prediction score for independent walking 1-year after TSCI is helpful in guiding patient management	3 (50%)	1 (16.7%)	1 (16.7%)	0	1 (16.7%)	6

Two respondents gave additional feedback on their experience using Ambulation.

Respondent 1: “*I think there should be clarification on the website what independent ambulation means. From my take on the Hicks paper, they used a 50 m distance with or without aids (i.e., FIM of 6 or 7), whereas Middendorp used 10 m as their distance on the SCIM. I have concerns that the individual prediction of a population may not fully represent real scenarios. The model really downgrades older people. For example, any older person with even minor spinal cord injury (e.g., grade 5 motor power and grade 1 sensation) is predicted not able to walk. I question the face validity of this*.”
Respondent 2: “*I trialed this tool using a patient who is now 2 years post TSCI. I used the ISNCSCI that was completed in ICU 2 days post injury. The patients score was 35.87%. The Ambulation Tool was correct in its prediction of independent ambulation. Even after a year of outpatient physiotherapy working on the patient's goal of ambulation this individual is not an independent ambulator*.”

### Google analytics data traffic

From February 7th, 2022 to February 28th, 2023, Ambulation had 594 total users, 565 (95.1%) new users, 26 (4.4%) returning users (Table 2). These refer to unique visits and repeat visits, respectively. In addition, there were 363 (61.1%) engaged sessions (i.e., the number of sessions that lasted 10 seconds/longer, or had one/more conversion events or two/more page or screen views). In terms of engagement with different pages, 213 users visited the Ambulation home page, and 164 users visited the calculation page. For specific tracking of the “calculate” and “re-calculate” buttons, 167 and 67 users were clicking these, respectively. The “calculate” button had an average event count of 1.29 times, and the “re-calculate” button had an average event count of 2.57 times, meaning that on average these 67 users pressed “re-calculate” at least 2.5 times.

**Table 2:** Ambulation google analytics data, report February 7, 2022 to February 28, 2023.

**Data variable**	**Data element definition**	***N* (%)**
Users	The total number of active users	594
New users	The number of users who interacted with your site or launched your app for the first time (event triggered: first_open)	565 (95.1)
Returning users	Users who have initiated at least one previous session	26 (4.4)
Users by country	The country from which the user activity originated	1. United States, 236 (39.9) 2. Canada, 226 (38.2) 3. China, 48 (8.1) 4. Saudi Arabia, 13 (2.2) 5. Australia, 10 (1.7)
Users by Canadian City	The city from which the user activity originated	1. Toronto, 53 (11.6) 2. Ashburn, 30 (6.6) 3. Columbus, 19 (4.2) 4. Ottawa, 18 (3.9) 5. Vancouver, 17 (3.7) 6. Hamilton, 16 (3.5) 7. Montreal, 15 (3.3)
Sessions—direct search by new users	This is most often the result of a user entering a URL into their browser or using a bookmark to directly access the site	411
Session—organic search	This refers to sessions from users who found the website via an organic search, i.e., they found the website after clicking on the website's link in the search engine results page	174
Event count	The number of times your users triggered an event	4,525
Event name by total users	The name of the triggered event	1. page_view, 5682. session_start, 5683. user_engagement, 3564. first_visit, 5655. scroll, 3256. Calculate button Click, 1677. Recalculate button click, 67
Average engagement time	Average engagement time per active user for the time period selected	53 s
Engaged sessions per user	Average session count per active user for the time period selected	0.61
Users by platform	The platform on which your app or website ran; e.g., web, iOS, or Android	Web, 594 (100)
Users by operating system	The operating systems used by visitors to your app or website. Includes mobile operating systems such as Android	1. Windows, 333 (56.1) 2. Macintosh, 123 (20.7) 3. iOS, 89 (15.0)
Users by browser	The browsers used to view the website	1. Chrome, 3972. Safari, 1133. Edge, 38
Users by device category	The type of device: desktop, mobile, or tablet	1. Desktop, 423 (79.4) 2. Mobile, 109 (20.5) 3. Tablet, 1 (0.2)

Users were primarily from the United States (236, 39.9%), Canada (226, 38.2%), and China (48, 8.1%), and were accessing Ambulation using a desktop (463, 77.9%) or mobile (130, 21.9%) device.

## Discussion

In an effort to bridge the KT gap for SCI prediction models, we implemented and designed a simple front-end website CPR for predicting independent walking ability 1-year after TSCI using the model by Hicks et al. (25). Ambulation was successfully developed, piloted, and launched. Within its first year, GA data demonstrated that Ambulation steadily gained new users over time. Peaks in new users coincided with presentations at local and international conferences. Overall, the majority of users originated from the United States and Canada. Of note, from the 234 unique users that eventually performed a calculation during the study period, 67 (28.6%) users clicked the re-calculate button on average >2.5 times (Table 2), suggesting further utility of the CPR beyond an initial calculation.

In addition to developing the CPR as an application, our experience with developing Ambulation demonstrates the importance of additional considerations when developing web-based KT tools. These include the engagement of professionals in IT, communications, marketing, privacy policy, and persons with lived experience along with targeted end-users, bringing a diverse array of knowledge and skill sets to help design appropriately for users' needs; and the inclusion of additional web pages to meet privacy requirements and support end-users. We engaged end-users as active participants across the design, development, and testing phases of Ambulation through user-centered design principles (33). This helped promote the usability and applicability of the web-based calculator in the clinical setting, for example, by better understanding the technologies available to them and their clinical workflows. Moreover, it was particularly important to precisely identify and engage with the targeted end-user in developing content for each of the web pages. This was needed to effectively tailor the information provided on the website. For example, the first question on the Home Page (Figure 1), “What is traumatic spinal cord injury?”, was kept brief assuming that clinicians using the website would already have some level of expertise on TSCI. Similarly, on the Calculator Page (Figure 2), resources for detailed information on how to perform the ISNCSCI assessment were provided. This would not be needed if, for example, the Ambulation calculator was designed for direct use by persons living with SCI. Defining and engaging the end-user early in the implementation process, a key component of the knowledge-to-action (KTA) cycle (12) and integrated KT (30), helps ensure that the tool being developed is ultimately relevant to users' needs.

The process of translating knowledge to practice is iterative and dynamic (12, 13). The development of Ambulation represents one phase of Knowledge Creation, which is part of the KTA cycle (34). Knowledge Creation involves (1) knowledge inquiry, (2) synthesis of knowledge, and (3) the production of knowledge tools. The methods described in this paper primarily encompass the third phase, with the creation of the web-based calculator as a KT tool, whereas the former two were accomplished with the development and validation of the CPR by Hicks et al. (25). While commentators have raised a variety of issues related to the translation of prognosis research into practice, a recurring theme is the lack of tools to simplify the complexity of prognostic models for daily use in clinical settings and the failure to recognize prognostic models as healthcare technologies that require deliberate implementation strategies (1, 2). These claims align with the current state of SCI research.

In a systematic review of KT initiatives in SCI research, Noonan et al. identified a paucity of KT interventions for SCI (14). Moreover, and perhaps unsurprisingly, none of the few interventions identified were related to implementing CPRs into clinical practice. As machine learning driven prognostic studies become more important in spine research (4), there will be a pressing need to create web-based tools that incorporate these complex models and make them more accessible to potential end-users, given the novelty of these techniques compared to traditional regression-based CPRs. Therefore, the focus on the production of knowledge tools presented here (Phase 3 of Knowledge Creation), addresses an important gap in the KTA cycle for prognosis research generally, and SCI prognostic models specifically. The development of CPRs in easy-to-use CPR presentation formats, such as Ambulation, could provide the means for implementing KT tools and better promote their adoption in clinical settings. We intended our approach to the design and development of Ambulation to be simple and with minimal resource requirements. This was done for two reasons: (1) to promote the reproducibility of our methods for other researchers seeking to develop web-based calculators alongside new CPRs and (2) to enable its transportability and integration with hospital-based systems in the future. While input from IT professionals and software engineers can be useful, the first steps to building web-based KT tools can be initiated by researchers with minimal technical expertise.

Concurrent with the pilot launch of Ambulation, several feedback mechanisms were integrated into the development of the website to provide means of evaluating the process of implementation and potential barriers or facilitators to the use of the CPR. These were the online feedback survey, the website traffic data collected through Google Analytics, and the Contact page where users could find information to email comments or suggestions. Through the survey, users provided feedback on how specific predictions provided through the calculator compared with their clinical intuition and experience. This provided insights about the face validity of the Hicks et al. (25) CPR. Encouraging this feedback is critical to the adoption of CPRs, if experts do not accept the results from the web-based calculator, they are less likely to adopt the tool and use it to inform discussions with patients. Future updates or refinements of the CPR to optimize its clinical usefulness, such as modifying the risk threshold for classifying patients as able to walk or not based on the estimate of risk (20), can be also implemented and tested in this way. The integrated GA metrics (Table 2) supplemented survey feedback by allowing continuous monitoring of user behavior on the website. Even with our small sample and limited study period, these feedback mechanisms revealed critical insights into the implementation process and provided data to the research team in real-time to facilitate improvements to the design of the web-based calculator. This iterative approach to implementation allows for early identification of usability issues, prompt redesign, and further testing—principles that promote the successful design and implementation of digital health innovations (33).

### Limitations

This study has several limitations. First, the response rate to the feedback survey was low (six of 30, 20%). However, the purpose of soliciting feedback was to assess usability issues and improve the overall design, thus achieving a reasonable number of responses (rather than a high rate of response) was our goal. Research suggests that as few as three to five users can identify the most important issues for usability testing (35, 36), therefore the six respondents may be adequate to provide baseline feedback for improving Ambulation. Second, test users were limited to clinicians in Canada. Engaging users from diverse healthcare settings could offer new learning points for design and implementation and improve the generalizability of Ambulation's implementation. Third, we did not assess which clinical settings and at which timepoints during the clinical workflow Ambulation was tested among users. Collecting these data will facilitate workflow analysis and help promote the adoption and sustained use of Ambulation by clinicians treating persons with TSCI over time. Lastly, the clinical utility of Ambulation is limited to the setting and populations in which the CPR by Hicks et al. (25) was developed and validated: adult patients with TSCI managed in acute care and rehabilitation hospitals across Canada (26).

### Future research directions

We presented the process of developing and piloting Ambulation, however, it is yet to be elucidated how to effectively integrate this web-based CPR into routine clinical practice. To do this, further engagement with the KTA cycle proposed by Straus et al. (12) is needed. This will entail evaluating the adoption or customization of the KT tool to the local context; assessing the determinants of use; and determining strategies for ensuring sustained use. This can be facilitated through conducting a focused implementation study guided by validated frameworks such as the Reach, Effectiveness, Adoption, Implementation, and Maintenance (RE-AIM) or Promoting Action on Research Implementation in Health Services (PARIHS) (37). Furthermore, there is an opportunity to develop other SCI web-based calculators using our experience from Ambulation, similar to the work being pursued by the SORG Orthopedic Research Group (https://sorg.mgh.harvard.edu/predictive-algorithms/), who have developed several predictive algorithms for patient outcomes after orthopedic surgery (38). Here, different tools have been made accessible for a variety of conditions and clinical decisions. For SCI, we plan to develop a library of web-based CPRs to bridge the KTA gap that covers the spectrum of clinical care including prediction of survival (39), functional capabilities besides walking (e.g., bowel and bladder function) (40, 41), life satisfaction, quality of life, and readmission or discharge disposition. These KT tools will align to the priorities of people with lived experience as well as clinicians and will focus on those developed or validated with Canadian data to ensure applicability in a Canadian context. Finally, future work should consider feedback from a larger group of clinicians from centers in other countries. The easy accessibility of Ambulation could also provide means for conducting external validation of the Hicks et al. CPR in under-researched settings (e.g., low-to-middle countries). The results of this study already demonstrate the use of this tool in three continents (Table 2). With further dissemination of the Ambulation website to both Low- and Middle-Income Countries (LMICs) and High-Income Countries (HICs), an emerging focus of future research may be comparative analysis of CPR utilization in diverse healthcare economies and the differences in therapeutic attitudes that these data may reflect.

In conclusion, Ambulation, a web-based CPR for independent walking 1-year after TSCI, was developed and successfully launched. Here we describe the steps to developing Ambulation and provide initial results from the pilot study among SCI clinicians. Feedback from the user survey suggests that clinicians believe Ambulation is useful in practice, easy-to-use, and may be of assistance for discharge planning. These findings outline some feasible options for developing web-based CPRs and some challenges that should be addressed to enable the implementation of CPRs in clinical care. We anticipate that our experiences with developing and launching Ambulation will promote and inform the development of other web-based presentation platforms and help improve future prediction model digital implementation efforts.

## Data availability statement

The datasets presented in this article are not readily available because of the Rick Hansen Spinal Cord Injury Registry (RHSCIR) Data Use and Disclosure Policy which is administered by the Praxis Spinal Cord Institute. Requests to access the datasets should be directed to the Praxis Spinal Cord Institute, dataservices@praxisinstitute.org.

## Ethics statement

Ethical review and approval was not required for the study on human participants in accordance with the local legislation and institutional requirements. Written informed consent from the patients/participants or patients/participants' legal guardian/next of kin was not required to participate in this study in accordance with the national legislation and the institutional requirements.

## Author contributions

RH, HH, NF, VN, and PP were involved with conceptualization, methodology, and project design. RH and HH developed and wrote the original draft, and prepared figures and tables. All authors revised the draft of the paper, contributed to the article, and approved the submitted version.
