# Supplementary material for: Enabling knowledge translation: implementation of a web-based tool for independent walking prediction after traumatic spinal cord injury
Source: Front Neurol. 2023 Dec 5;14:1219307. doi: 10.3389/fneur.2023.1219307 (PMC10728823; doi:10.3389/fneur.2023.1219307)
Supplement: Supplementary file 1 [file Table_1.DOCX]

# Appendix

**Supplementary Table S1**. The Ambulation User Survey

**Please tell us what you think about Ambulation. We welcome your feedback!**

**Q1. Please respond to each of the following statements, by selecting one response.**

|  | **Strongly agree** | **Somewhat agree** | **Neither agree, nor disagree** | **Somewhat disagree** | **Strongly disagree** |
| --- | --- | --- | --- | --- | --- |
| Ambulation is easy to navigate |  |  |  |  |  |
| Ambulation's links are easy to identify |  |  |  |  |  |
| Ambulation's design, layout, color and contrast are visually appealing |  |  |  |  |  |
| Ambulation, the calculation page is easy to use |  |  |  |  |  |
| Ambulation, the result generated is easy to understand |  |  |  |  |  |
| Ambulation, the clinical algorithm is easy to understand |  |  |  |  |  |
| Ambulation is applicable for clinical use |  |  |  |  |  |
| Ambulation's prediction score for independent walking 1-year after TSCI is helpful in guiding patient management |  |  |  |  |  |

**Q2. Which are you more likely to use Ambulation on?**

Desktop PC/Mac

Smartphone device

**Q3. Would you recommend Ambulation to others?**

Yes, please specify why

No, please specify why

|  |
| --- |
|  |

**Q4. Please provide any additional feedback about your experience with Ambulation**

|  |
| --- |
|  |

**Thank you for your feedback!**
